# Syphilis: A Forgotten Priority

**DOI:** 10.1371/journal.pmed.0030204

**Published:** 2006-04-25

**Authors:** Damian Walker, Godfrey Walker

**Affiliations:** **1**London School of Hygiene and Tropical MedicineLondonUnited Kingdom; **2**Johns Hopkins UniversityBaltimore, MarylandUnited States of America; **3**United Nations Population FundNew York, New YorkUnited States of America

Peter Hotez and colleagues [ 1] provide a persuasive case for incorporating a rapid-impact package for “neglected tropical diseases” with programs for HIV/AIDS, tuberculosis, and malaria as part of a pro-poor strategy for improving health in the developing world. However, we believe there is a disease that has a high claim to be included in partnerships and initiatives devoted to what the authors term the “Big Three”, and yet has been largely ignored.

On the basis of the criteria identified by Hotez et al., the case for giving explicit priority to programs to control syphilis and particularly congenital syphilis is high [ 2]. In 2002, there were 157,000 deaths contributing to more than 4 million disability-adjusted life years (DALYs) (see annex tables 2 and 3 in [ 3]). These estimates exclude the burden attributable to maternal syphilis, which includes 460,000 abortions or stillbirths, 270,000 low-birth-weight babies, and 270,000 cases of congenital syphilis each year [ 4]. This burden is concentrated in Africa and exhibits considerable geographic overlap with HIV infection. Syphilis accounts for 20% of genital ulcer diseases and is a cofactor in transmission of HIV, and both infections appear to progress more rapidly when they occur together [ 5].

Infection with syphilis is curable, and control is possible with existing drugs (specifically penicillin). However, little attention has been given to this in context of the Big Three. Azithromycin is included in the chemotherapy package proposed by Hotez et al. for the control of trachoma, and there are clear synergies with syphilis control. A recent trial in Tanzania demonstrated that a single dose of oral azithromycin is as effective as injectable penicillin G benzathine in treating early and latent syphilis [ 6]. However, some caution is needed concerning the widespread use of azithromycin for syphilis in view of the recent emergence of azithromycin-resistant Treponema pallidum [ 7].

There are other possible synergies in having a strategy including syphilis control; e.g., during routine antenatal care, chemotherapy for soil-transmitted helminths could be provided at the same time as offering voluntary counselling and testing (VCT) for HIV infection and screening for maternal syphilis. The control of syphilis has been shown to be highly cost-effective. If the control of syphilis was integrated into programs dealing with the four priority disease groups advocated by Hotez et al., then the cost-effectiveness of tackling not only syphilis but also the other four major public health priorities would improve. Furthermore, it would lessen the chance of avoiding death from one disease but dying from another [ 8].

While it might be hoped that the case for giving priority to syphilis would have been accepted and explicit emphasis given to programs to control this disease, this has not happened. Unfortunately, limited attention is given to syphilis control as part of the several partnerships devoted to the Big Three. Maybe this is because syphilis has historically had a social stigma, and has, therefore, been neglected. Now is the time to change this as part of a pro-poor strategy to meet the Millennium Development Goals. We suggest it is explicitly included in the rapid-impact package for neglected tropical diseases.
